# Systematic review and meta-analysis of Murray’s law in the coronary arterial circulation

**DOI:** 10.1152/ajpheart.00142.2024

**Published:** 2024-05-24

**Authors:** Daniel J. Taylor, Harry Saxton, Ian Halliday, Tom Newman, D. R. Hose, Ghassan S. Kassab, Julian P. Gunn, Paul D. Morris

**Affiliations:** ^1^Division of Clinical Medicine, School of Medicine and Population Health, University of Sheffield, Sheffield, United Kingdom; ^2^Materials and Engineering Research Institute, Sheffield Hallam University, Sheffield, United Kingdom; ^3^Insigneo Institute for In Silico Medicine, University of Sheffield, Sheffield, United Kingdom; ^4^Department of Cardiology, Sheffield Teaching Hospitals NHS Foundation Trust, Sheffield, United Kingdom; ^5^NIHR Sheffield Biomedical Research Centre, Sheffield Teaching Hospitals NHS Foundation Trust, Sheffield, United Kingdom; ^6^California Medical Innovations Institute, San Diego, California, United States

**Keywords:** bifurcation, left main coronary artery, Murray’s law

## Abstract

Murray’s law has been viewed as a fundamental law of physiology. Relating blood flow ($\dot{\text{Q}}$) to vessel diameter (*D*) ($\dot{\text{Q}}$·∝·*D*^3^), it dictates minimum lumen area (MLA) targets for coronary bifurcation percutaneous coronary intervention (PCI). The cubic exponent (3.0), however, has long been disputed, with alternative theoretical derivations, arguing this should be closer to 2.33 (7/3). The aim of this meta-analysis was to quantify the optimum flow-diameter exponent in human and mammalian coronary arteries. We conducted a systematic review and meta-analysis of all articles quantifying an optimum flow-diameter exponent for mammalian coronary arteries within the Cochrane library, PubMed Medline, Scopus, and Embase databases on 20 March 2023. A random-effects meta-analysis was used to determine a pooled flow-diameter exponent. Risk of bias was assessed with the National Institutes of Health (NIH) quality assessment tool, funnel plots, and Egger regression. From a total of 4,772 articles, 18 were suitable for meta-analysis. Studies included data from 1,070 unique coronary trees, taken from 372 humans and 112 animals. The pooled flow diameter exponent across both epicardial and transmural arteries was 2.39 (95% confidence interval: 2.24–2.54; I^2^ = 99%). The pooled exponent of 2.39 showed very close agreement with the theoretical exponent of 2.33 (7/3) reported by Kassab and colleagues. This exponent may provide a more accurate description of coronary morphometric scaling in human and mammalian coronary arteries, as compared with Murray’s original law. This has important implications for the assessment, diagnosis, and interventional treatment of coronary artery disease.

## Graphical abstract

**Figure:**
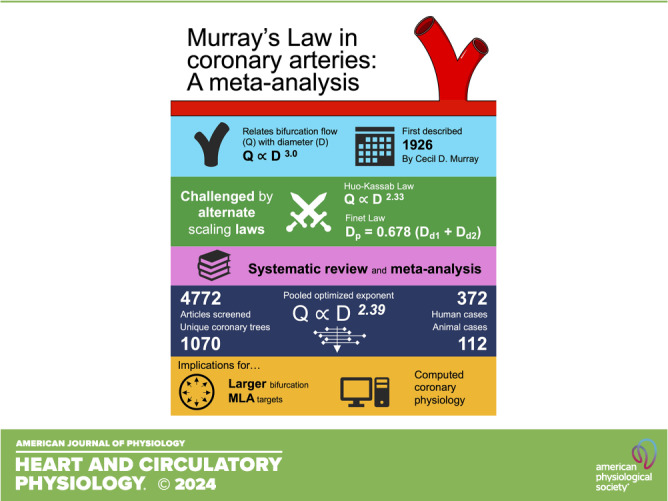

## INTRODUCTION

First described in 1926, Murray’s law (1) is a fundamental principle of biology that relates the form and function of all branched transport networks. Derived from the principal of minimum work, it characterizes the equipoise between the energy required to produce and maintain blood volume against that required to overcome viscous friction. In its simplest form, flow ($\dot{\text{Q}}$) is proportional to the cube of vessel diameter (*D*) ($\dot{\text{Q}}$·∝·*D*^3^). Assuming conservation of mass, Murray’s law also characterizes the relationship between the diameters of the parent vessel (PV) and daughter vessel (DV) around bifurcations (${D_{\text{PV}}}^{3}\;=\;{D_{\text{DV1}}}^{3}\;+\;{D_{\text{DV2}}}^{3}$). Murray’s law, therefore, has most biological relevance to the epicardial and transmural coronary arteries, whose main purpose is the transportation of blood. This contrasts with the perfusing vessels of the distal tree, where rapid expansion of cross-sectional area (i.e., the flow-diameter scaling exponent) facilitates deceleration of blood and effective substrate exchange.

Given the ubiquitous nature of Murray’s law, its importance for our understanding of both vascular physiology and implications for clinical medicine are far-reaching (2–4). This is particularly true in clinical cardiology, where Murray’s law has become synonymous with the coronary arterial circulation, defining the appropriate size of coronary arteries within a bifurcation. Indeed, international guideline documents (5, 6) base the indications and targets for percutaneous coronary intervention (PCI) on minimum lumen area (MLA) criteria derived directly from Murray’s law and validated in a landmark clinical trial (7). Murray’s law has also found application in computational fluid dynamics (CFD) modeling of the coronary vasculature, where clinical tools that predict the physiological significance of coronary artery disease use the relationship between anatomy and physiology to determine flow splitting at bifurcations. Such CFD techniques may be applied to both CT (8) and invasive angiography modalities (9, 10) and are rapidly being adopted in routine clinical practice (11). Furthermore, the flow-diameter exponent may affect diagnostic accuracy (12, 13).

**Figure 1.**
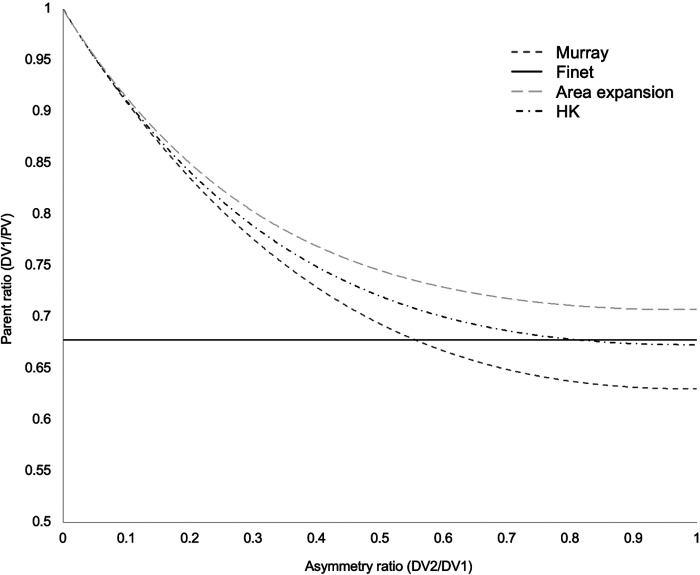
Agreement between scaling laws as a function of asymmetry. Note the close agreement between the laws of Huo and Kassab (17) and Finet et al. (18) for highly asymmetrical bifurcations.

Murray’s law assumes steady, laminar flow of a Newtonian fluid in isolated bifurcations. These criteria are not necessarily satisfied within the coronary circulation, which has implications for the flow-diameter exponent. When turbulent, unsteady flow and the rheological properties of blood are accounted for, an exponent between 2.0 and 3.0 is retrieved (14–16). Unlike Murray’s original work, which considered each bifurcation in isolation, Huo and Kassab (HK) (17) considered resistance of the entire vascular tree, which lies distal to each daughter branch in a bifurcation, to derive a reformulated HK law, with a flow-diameter exponent of 7/3 (i.e., 2.33). Finally, the law with perhaps most clinical recognition is that of Finet (18), who used intravascular ultrasound (IVUS) data, taken from 173 major epicardial bifurcations, to parameterize a fractal relationship of bifurcation morphology [*D*_PV_ = 0.678 (*D*_DV1_ + *D*_DV2_)].Differences in these morphometric scaling laws generate inconsistency in the predicted parameters of coronary bifurcations, which is also dependent upon daughter vessel asymmetry (Fig. 1). The clinical implications of uncertainty in the optimal flow-diameter scaling exponent are important. Compared with other recognized theoretical exponents, a Murray’s exponent of 3.0 will underestimate MLA targets by as much as 20% (19). Despite considerable research, the optimum flow-diameter exponent for coronary arteries remains unknown. The aim of this meta-analysis was to quantify the optimum flow-diameter exponent in the epicardial and transmural mammalian coronary arterial tree.

## METHODS

We followed the Preferred Reporting Items for Systematic Reviews and Meta-Analyses (PRISMA) (20) and Meta-analysis of Observational Studies in Epidemiology (MOOSE) (21) reporting guidelines in performing this systematic review and meta-analysis (Supplemental Tables S1 and S2; all supplemental materials may be found at https://doi.org/10.7910/DVN/PIL9NE), which was also registered with PROSPERO (Registration No. CRD42023416529).

### Search Strategy

The following databases were searched for published studies in peer-reviewed journals from inception to 20 March 2023: Cochrane library, PubMed/Medline, Scopus, and Embase. The following keywords were used: Murray law, Huo Kassab law, Finet law, scaling law, flow radius, flow diameter, diameter ratio, power law, exponent, Murray ratio, area expansion ratio, volume length, diameter length, radius length, area length, and coronary (see Supplemental Table S3 for full search criteria). The wildcard term (*) was used to increase search strategy sensitivity. Because of widespread heterogeneity of nomenclature for the flow-diameter exponent in the literature, all articles indexed in the Web of Science platform referencing the original Murray's law paper (1) and including the term “coronary,” in addition to articles identified through citation chasing, were also included.

### Article Eligibility

Articles were considered eligible for meta-analyses if they reported an optimum flow-diameter exponent in whole/subsections of mammalian epicardial or transmural coronary trees (i.e., not perfusion arterioles) with associated standard error or standard deviation and number of included participants. If data were unclear or reported only in figures, corresponding authors were contacted through email to seek clarification/raw data. If corresponding authors could not be contacted, data extraction was performed from figures with the online software WebPlotDigitiser (V.4.6). Accuracy of plot digitization was verified with a randomly generated dataset (Supplemental Table S4). In all instances where exponent standard error was retrospectively calculated from digitized plots, methods are reported in Supplemental Table S5. Articles not suitable for meta-analyses were still included in the report if they reported an optimum flow-diameter exponent in mammalian conducting coronary trees. Articles were excluded if they reported a flow-diameter exponent not specific for coronary arteries, were unpublished, or not published in the English language. Conference abstracts were not considered.

Study selection was performed independently by three investigators (D.J.T., P.D.M., and I.H.), who are a mix of clinical academics (D.J.T. and P.D.M.) and a computational modeling professor (I.H.). Titles and abstracts were assessed by D.J.T. and P.D.M., studies deemed suitable for inclusion by one or both investigators were included for full-text screening. D.J.T., P.D.M., and I.H. performed full-text screenings, with discrepancies resolved by consensus. Data for all compatible results were extracted and stored in a preformatted spreadsheet, which included data on species, disease state, size of coronary arteries studied (diameter or Strahler order), and the optimal flow diameter exponent (Supplemental Table S6). Plot digitization was performed by D.J.T. and verified by P.D.M. and I.H. For each study included in the meta-analyses, risk of bias was assessed independently by D.J.T. and P.D.M. using the National Heart, Lung, and Blood Institute observational cohort and cross-sectional study quality assessment tool and used to rate studies as “good,” “fair,” or “poor” (22). For studies whose authorship posed a conflict of interest in bias assessment, a third reviewer (H.S.) independently performed this analysis.

### Data Synthesis and Statistical Analysis

The primary end point was the pooled optimized flow-diameter exponent for the conducting portion of mammalian coronary trees (vessel Strahler order ≥5). Secondary outcomes included the pooled flow-diameter exponent for humans and animals separately, for epicardial versus transmural coronary arteries and the pooled exponent in subjects with versus without cardiovascular disease. The meta-analysis was computed using the Meta-Essentials software package (V.1.5, Erasmus Research Institute of Management) (23). For studies reporting exponents with a skewed distribution, log-transformation was applied, provided this satisfied a log-normal assumption. Results were then combined such that all were comparable on an absolute scale (24). As some studies reported multiple exponents from the same clinical dataset, an analysis was performed to quantify sensitivity to study weighting. For this sensitivity analysis, a prior intrastudy meta-analysis was performed to derive a single flow-diameter exponent for each study, which was then used for pooled interstudy comparisons. A fixed-effect model was used for intrastudy meta-analysis, as between-group variability could not be efficiently quantified (25, 26). The standard formula for pooled estimates was used to calculate μ and σ (27). Interstudy heterogeneity was assessed using Cochrane’s $\dot{\text{Q}}$ test and the I^2^ index. When significant heterogeneity between studies was present (I^2^ > 50%), the interstudy-pooled flow-diameter exponent was calculated with a random-effects model (28). When heterogeneity was not significant, a fixed-effects model was used. Tabulated results from all studies reporting an optimized flow-diameter exponent are presented, with results of the interstudy meta-analysis also displayed graphically with forest plots. Risk of reporting bias was assessed using the National Institutes of Health (NIH) quality assessment tool. Publication bias was assessed with funnel plots and Egger regression.

## RESULTS

### Study Selection

**Figure 2.**
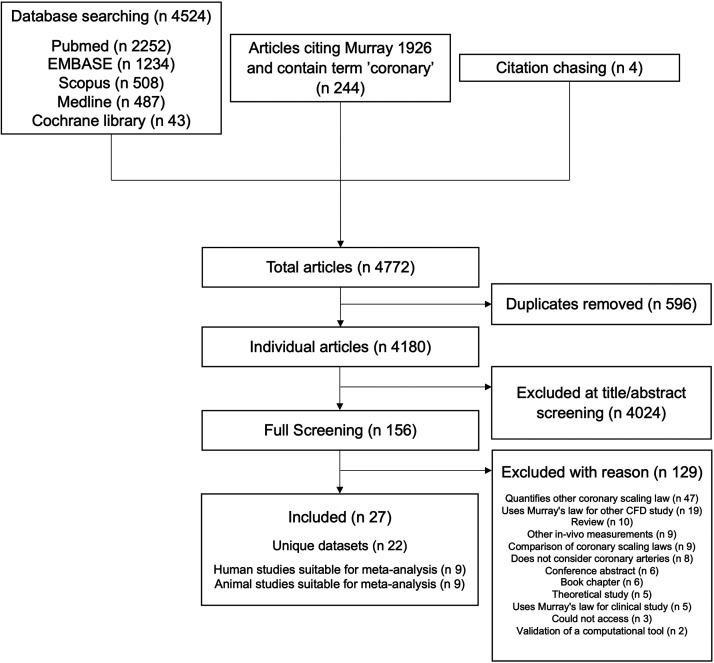
Consort diagram.

A total of 4,524 articles were identified through database searching, 244 by searching articles citing the original Murray’s law paper and 4 through citation chasing. After duplicate removal, 4,180 unique articles were identified. Title/abstract screening removed 4,024 articles, leaving 156 articles for full-text screening. Full-text screening identified 27 articles quantifying a flow-diameter exponent (Supplemental Table S6). These articles were based upon 22 unique datasets, as 6 articles (29–34) analyzed the same morphometric data from 5 pigs originally described by Kassab et al. (35). Of these 22 studies, 18 were suitable for meta-analysis (Fig. 2).

Eleven studies analyzing the flow-diameter relationship of human coronary arteries were identified, of which nine were included in meta-analyses. These nine studies included data from 372 individuals and 826 unique bifurcations/arteries. Most studies (*n* = 6) conducted retrospective analysis of clinical data taken from healthy coronary arteries during clinically indicated evaluation for ischemic heart disease (19, 36–40). One study quantified exponents in epicardial arteries with a mix of healthy (*n* = 42) and diseased (*n* = 68) participants (41), whereas the remaining two studies each analyzed a single, healthy heart postmortem (42, 43). The two studies not suitable for meta-analysis included one CT coronary angiography (CTCA) analysis of nondiseased left epicardial vessels in 211 participants, reporting an exponent of 2.4 (44), whereas the second study conducted whole tree analysis of the left coronary circulation of a nondiseased single human heart, reporting an optimal exponent of 2.53, increasing to 3.21 in the smallest vessels (45) (Table 1).

**Table 1.** Studies quantifying an optimal flow-diameter exponent in human coronary arteries

Study	Participants, *n*	Cardiovascular Pathology	Vessels Analyzed	Exponent ± SD/(95% CI)
Suwa et al. (43)	1	Not reported	≥100 μm	2.51 ± 0.084
			<100 μm	2.82 ± 0.097
Hutchins et al. (41)	42	AS0	LMCA bifurcation	3.2 ± 1.6
	26	AS1	LMCA bifurcation	2.8 ± 1.3
	25	AS2	LMCA bifurcation	2.6 ± 1.5
	17	AS3/4	LMCA bifurcation	2.2 ± 2.1
	53	AS0	Non-LMCA epicardial bifurcations	2.7 ± 1.3
Changizi and Cherniak (36)	33	Nondiseased	Epicardial bifurcations	2.60 ± 0.64
Zamir (42)	1	Nondiseased	RCA bifurcations >1.0 mm	2.01 ± 0.78
Ellwein et al. (37)	55	Diameter stenosis >50% excluded	LMCA bifurcation	2.67 (2.25–3.16)
		Diameter stenosis >50% excluded	LAD bifurcation	1.28 (1.15–1.43)
		Diameter stenosis >50% excluded	LCx bifurcation	1.14 (1.00–1.31)
Medrano-Gracia et al. (44)	211	Nondiseased	Epicardial bifurcations	2.4
van der Giessen et al. (38)	6	Nondiseased	Epicardial bifurcations	Flow diameter fit: 2.55 (2.27–2.83)
				Flow ratio fit: 2.27 (1.58–2.96)
Choi et al. (40)	43	Nonobstructive CAD	Epicardial arteries	2.27 ± 0.24
Blanco et al. (39)	50	Nondiseased	LMCA bifurcations	Anatomic fit: 2.32 ± 1.05
				Simulation fit: 2.62 ± 0.64
Taylor et al. (19)	20	Nondiseased	Epicardial arteries	Flow fit: 2.15 (1.38–3.20)
				*R*_micro_ fit: 2.38 (1.34–3.36)
Schwarz et al. (45)	1	Atrial tachycardia, mitral stenosis, atherosclerosis	Epicardial, 5.0 μm	2.53

All measurements represent vessel diameter. AS, artery stenosis grade; CAD, coronary artery disease; CI, confidence interval; INOCA, ischemia with nonobstructive coronary artery; LAD, left anterior descending artery; LCx, left circumflex artery; LMCA, left main coronary artery; RCA, right coronary artery, *R*_micro_, microvascular resistance.

Sixteen articles quantified an optimal flow-diameter exponent in mammalian animal coronary arteries. These studies analyzed 11 unique datasets, as 6 (29–34) analyzed the same morphometric data of 5 pigs originally described by Kassab et al. (35) (Table 2). Nine articles were included in the meta-analysis, comprising morphometric data from 244 unique coronary trees from 53 pigs, 44 rats, 11 mice, and 4 dogs. Most studies (*n* = 7) included transmural vessels, whereas two studies (46, 47) examined the epicardial vessels exclusively. One study investigated the effects of ventricular hypertrophy on flow-diameter exponent (48), while all other studies examined only healthy vessels. The 7 studies not suitable for meta-analysis (29–32, 49–52) quantified exponents in 15 dogs, 5 unique pigs, and the 5 pigs described by Kassab et al. (35); exponents ranged from 2.06 to 3.50 (Table 3).

**Table 2.** Summary of studies reporting exponents from morphometric data of 5 pigs, originally described by Kassab et al. (35)

Study	Vessel Diameter	RCA	LAD	LCx	Combined
Zhou et al. (30)	Epicardial, 500 μm	2.18	2.21	2.51	
Mittal et al. (29)	Epicardial, ≤8 μm	2.2	2.1	2.1	
Kassab (31)	Epicardial, ≤8 μm	2.18	2.18	2.06	
Kassab (32)	Epicardial, ≤8 μm	2.09	2.10	2.10	
Kaimovitz et al. (33)	Epicardial (vessel order 11-8)	1.86†	1.99†	1.88†	1.90†
	Transmural (vessel order 7-5)	1.48†	1.43†	1.65†	1.54†
Sturdy et al. (34)	Epicardial				2.47

LAD, left anterior descending artery: LCx, left circumflex artery; LMCA, left main coronary artery; RCA, right coronary artery.

†Digitally extracted data and combined through fixed effects meta-analysis.

**Table 3.** Summary of animal studies quantifying an exponent, excluding pigs described by Kassab et al. (35)

Study	Subjects, *n*	Vessels Analyzed	Exponent ± SD
Arts et al. (47)	Dogs (9)	Epicardial, 400 μm	2.55 ± 0.03
VanBavel and Spaan (49)	Pigs (2)	Epicardial, >200 μm	2.35
		200–40 μm	2.50
		<40 μm	2.82
Zhou et al. (46)	Pigs (5)	LAD, 500 μm	2.71
Tomanek et al. (51)	Dogs (14)	50–9 μm	2.73
Gong et al. (48)	LVH pigs (6)	Epicardial	2.51 ± 0.48
		Transmural	2.07 ± 0.14
	RVH pigs (6)	Epicardial	2.58 ± 0.65
		Transmural	2.15 ± 0.14
	CHF pigs (6)	Epicardial	3.15 ± 1.49
		Transmural	2.26 ± 0.15
	LVH control (6)	Epicardial	2.62 ± 0.74
		Transmural	2.01 ± 0.12
	RVH control (6)	Epicardial	2.39 ± 0.73
	Transmural	1.95 ± 0.13
	CHF control (6)	Epicardial	2.69 ± 0.69
		Transmural	2.10 ± 0.13
Rivolo et al. (50)	Pig (3), Dog (1), Human (1)	Epicardial, 100 μm	∼2.25 to ∼3.5
Li et al. (53)	Mice (11)	Epicardial, 40 μm	2.26 ± 0.26
Zamir et al. (54)	Pigs (7)	Epicardial, 40 μm	3.53†
Wieringa et al. (55)	Rats (38)	37–15 μm	2.81
Demeulenaere et al. (56)	Rats (6)	150–10 μm	2.61

CHF, congestive heart failure; LAD, left anterior descending artery; LCx, left circumflex artery; LVH, left ventricular hypertrophy; RCA, right coronary artery; RVH, right ventricular hypertrophy. All vessel measurements represent diameter. †Digitally extracted data and combined through fixed effects meta-analysis.

### Risk of Bias and Publications Bias

Risk of bias in the included studies showed that quality of most studies was generally fair or good (Supplemental Table S7). The highest risk of bias was seen in studies that failed to adequately describe the characteristics of included participants or account for large proportions of participant noninclusion. Several human studies did not adequately describe the patient population and attrition rates. Only a single study performed prior power analysis (39), whereas only one other attempted to control for confounding factors (48). There was no evidence of systematic reporting bias in flow-diameter exponent (Egger test, *P* = 0.15) (see Supplemental Fig. S1).

### Outcomes

**Figure 3.**
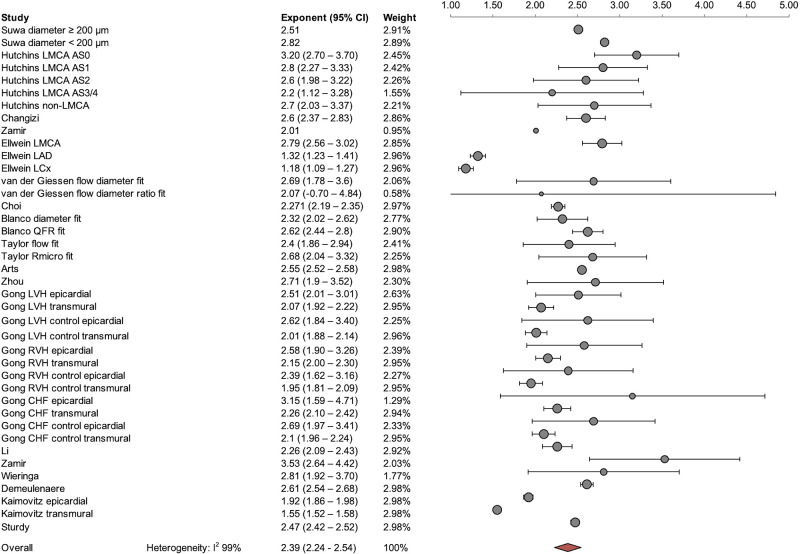
Meta-analysis of pooled flow-diameter exponent.

An optimal flow-diameter exponent was reported in 18 studies, including 489 total participants. The pooled flow-diameter scaling exponent across all participants was 2.39 [95% confidence interval (CI): 2.24–2.54] (Fig. 3). Between-study heterogeneity was large (I^2^ = 99%). The pooled exponent was relatively insensitive to reduced weighting of studies reporting multiple optimized flow-diameter exponents for the same clinical dataset (pooled exponent: 2.39; 95% CI: 2.19–2.56). The flow-diameter scaling exponent for humans and animals separately was 2.42 (95% CI: 2.17–2.67) and 2.36 (95% CI: 2.17–2.55). Overlap in exponent 95% CI ranges indicated no significant difference between epicardial and transmural vessels in exponent value: 2.43 (95% CI: 2.25–2.61) versus 2.21 (95% CI: 1.93–2.49), respectively. All cause cardiovascular disease also did not significantly alter the flow-diameter scaling exponent compared with healthy participants [2.29 (95% CI: 2.10–2.49) vs. 2.38 (95% CI: 2.19–2.56), respectively] (see Supplemental Fig. S2, *A*–*G*). Data supplements can be accessed here: https://doi.org/10.7910/DVN/PIL9NE.

## DISCUSSION

In our meta-analysis of the flow-diameter exponent in coronary arteries, we found a pooled exponent of 2.39 for the mammalian coronary tree. Of the theoretically derived laws, our findings appear to best corroborate the HK exponent of 7/3. This is reassuring, given the theoretical work that considers turbulence (15), rheological blood properties (16), and flow pulsatility (14), as seen in conducting epicardial and transmural coronary arteries, which also all suggest that an exponent of 3.0 is an overestimation. The similarity in results between humans and animals was expected; the underlying physics governing the flow in both groups should be comparable. The pooled exponent is also consistent with studies unsuitable for inclusion in meta-analysis (44, 49, 50). In addition, confidence intervals for the pooled exponent support other analyses that have suggested that Murray’s exponent of 3.0 is an overestimate (57–59) and those reporting the area-preservation exponent of 2.0 are an underestimate (60–62). Unlike Murray’s original law, our pooled exponent of 2.39 implies that wall shear stress (WSS) is not conserved uniformly throughout the coronary tree and is instead sensitive to vessel diameter (WSS·∝·*D*^−0.75^). Historically, a time-averaged WSS of ∼1.5 Pa was thought to be optimal for healthy human coronary arteries (63). The current meta-analysis results support more recent work proposing that the atheroprotective range for WSS for any given coronary tree ranges between 1.0 and 7.0 Pa (64). The biological and physical mechanisms underlying this variation in WSS are beyond the scope of the present, clinically focused study. However, our pooled exponent of 2.39 shows closest agreement with the HK scaling law and associated exponent of 2.33 (17). Similar to the original work of Murray (1), the HK law is based upon a minimum energy hypothesis, encapsulated in a ratio of viscous to metabolic dissipation, but extended to the whole coronary tree distal to a particular vessel. This HK model derives power law relationships between structure and function parameters, which have been validated experimentally (31) and shows excellent agreement with Finet’s independent, heuristic fractal scaling laws for typical epicardial arteries (18).

### Clinical Relevance

**Figure 4.**
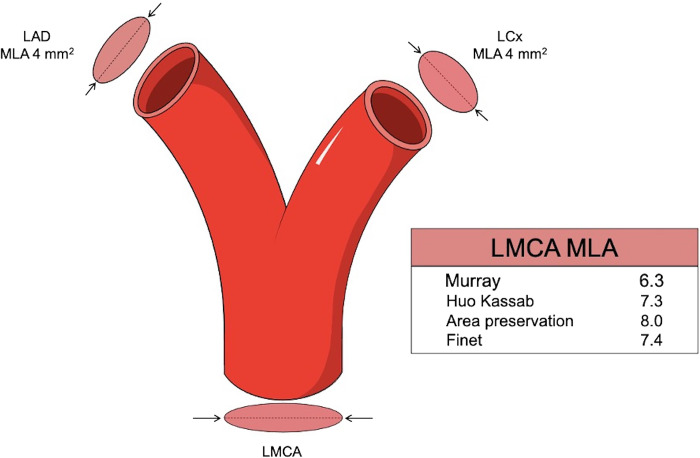
Variation in minimum lumen area (MLA) thresholds for left main coronary artery (LMCA) due to different theoretical exponents.

In clinical cardiology, the optimal flow-diameter exponent is relevant for the diagnosis and treatment of patients with suspected ischemic heart disease. Coronary intervention is focused entirely on restoring arterial diameter to allow sufficient myocardial blood flow. For nearly 100 years, our understanding of the relationship between arterial diameter and blood flow has been dictated by Murray’s law. European (6) and American (5) guidelines, along with the Bifurcation Academic Research Consortium (Bif-ARC) (65), recommend an MLA threshold derived from Murray’s law. These guidelines advocate for a left main coronary artery (LMCA) MLA of 6–7.5 mm^2^. The lower value of 6 mm^2^ was derived using a Murray’s exponent of 3.0 and prospectively validated in the multicenter, prospective LITRO study (7). Although this study showed that deferral of revascularization in LMCA lesions with an MLA < 6 mm^2^ was safe, use of the pooled exponent of 2.39 would have derived an MLA threshold of 7.1 mm^2^ (Fig. 4). The latter of these values shows closer agreement with observational data from 121 patients with angiographically normal or minimally diseased left coronary arteries (66) that informs the 7.5-mm^2^ upper MLA threshold. No studies have assessed the outcomes with higher minimum MLA thresholds. Furthermore, CFD techniques for evaluating virtual fractional flow reserve (FFR) of epicardial lesions use the flow-diameter exponent for determining proportion of flow splitting at bifurcations (8, 9, 19, 67, 68). The magnitude of exponent used has significant impact on diagnostic accuracy of virtual FFR for CFD techniques using both CT (13) and plane angiographic imaging data (12). Incorporation of flow-diameter scaling in virtual FFR workflows improves clinical utility, with very close agreement between single-view and three-dimensional reconstructions (69). Finally, in line with HK analysis (17), our pooled exponent gives a crown flow resistance parameter (ε) of 2.770, which may be used to describe interrelated diameter, length (*L*), and volume (V) scaling laws specific to the coronary tree:

$$D=L^{\frac{3\varepsilon \;-2}{4(\varepsilon +1)}}\;\text{V}=\;L^{\frac{5}{\varepsilon +1}}$$

These additional laws may aid in the diagnosis of diffuse coronary disease, which is often difficult with traditional techniques (70).

### Limitations

Heterogeneity was high (I^2^ = 99%), but this was expected, given the relatively low resolution of several imaging techniques used for quantifying coronary morphology and the well-documented challenges of assessing intracoronary flow (71). Nevertheless, this heterogeneity may represent a more subtle relationship between vessel diameter, resistance, and flow (30), which the current study is underpowered to detect. The exponents of 1.32 and 1.18 that were reported by Ellwein et al. (37) were a key source of heterogeneity. These results imply a marked acceleration of blood, which has not been observed, and so these results should be treated with caution. Limitations in data reporting did not allow for detailed quantification of the effect of cardiovascular disease on the flow-diameter exponent or an analysis of other variables of interest. Significant variability and the absence of consensus in the nomenclature of the flow-diameter scaling exponent meant the search strategy was not guaranteed to be completely exhaustive, necessitating the incorporation of citation chasing as an additional searching technique. Thresholds for discrimination between epicardial and transmural arteries may have differed between some studies, but as there was no difference in the exponent between the two vessel types, this is unlikely to be significant. Analysis of the effect of disease on the flow-diameter scaling exponent was limited by a small number of studies and disagrees with other findings reporting coronary artery disease (72), and lesion calcification (73) may influence the exponent. This may, therefore, be a topic for future research. The clinical utility of defining variations in the flow-diameter exponent for patient groups and vessel sizes is uncertain.

### Conclusions

In this meta-analysis, we identified an optimal flow-diameter exponent for Murray’s law in mammalian coronary arteries of 2.39. This is in very close agreement with the theoretically derived HK exponent of 7/3, which may be a more accurate description of coronary morphometric scaling compared with Murray’s original law. This finding may have implications for the assessment, diagnosis, and intervention of coronary artery disease.

## DATA AVAILABILITY

Data will be made available upon reasonable request.

## SUPPLEMENTAL DATA

10.7910/DVN/PIL9NE Template data collection, data extracted from included studies, and data used for all analyses are provided in Supplemental Tables S1–S7 and Supplemental Figs. S1–S2: https://doi.org/10.7910/DVN/PIL9NE.

## DISCLAIMERS

The views expressed are those of the authors and not necessarily those of the National Institute for Health and Care Research or the Department of Health and Social Care.

## DISCLOSURES

No conflicts of interest, financial or otherwise, are declared by the authors.

## AUTHOR CONTRIBUTIONS

D.J.T. and P.D.M. conceived and designed research; D.J.T., I.H., and P.D.M. performed experiments; D.J.T., H.S., I.H., G.S.K., J.P.G., and P.D.M. analyzed data; D.J.T., H.S., T.N., D.R.H., G.S.K., J.P.G., and P.D.M. interpreted results of experiments; D.J.T. prepared figures; D.J.T. drafted manuscript; D.J.T., H.S., I.H., T.N., D.R.H., G.S.K., J.P.G., and P.D.M. edited and revised manuscript; D.J.T., H.S., I.H., T.N., D.R.H., G.S.K., J.P.G., and P.D.M. approved final version of manuscript.
